# Supplementary material for: Development of a computer-aided design software for the quantitative evaluation of aesthetic damage
Source: PLoS One. 2019 Dec 18;14(12):e0226322. doi: 10.1371/journal.pone.0226322 (PMC6919621; doi:10.1371/journal.pone.0226322)
Supplement: S1 Code site — 1. (ZIP) [file pone.0226322.s001.zip › [EESC_jr][Projeto]Relatorio_07_21/reports/2/2017.07.04/09:20:09/report.pdf]

Preâmbulo

Na data de 04/07/2017 no horário 08:00, o(a) Perito(a) Professor Doutor Nelson Sakaguti, Cirurgião Dentista, designado(a)para proceder ao exame pericial solicitado em: luiz dalton ramos, do sexo Masculino, nascido(a) em 02/04/1985, ocupação pintor, portador(a) do RG 2345 ssp, cujo endereço caveira 2, passa a expor a avaliação de dano estético corporal.

Exame objetivo da lesão

A lesão ora objeto de análise teve como natureza Acidente de trabalho ocorrido em 21/05/2016. O prejuízo estético consiste em lesão cicatricial consolidada na região: Nariz, Bochecha no lado esquerdo.

Trata-se de cicatriz Hipertrófica de tamanho/frequência Múltipla: Complexa de orientação Horizontal de pigmentação Hipercrômica medindo 3 centímetro(s) na horizontal, 4 centímetros na vertical e 2 milímetros de Altura. De contornos Irregulares. A lesão tem repercussão é visível a 50 centímetros nas relações íntimas. A lesão é visível a 3 (três) metros nas relações sociais. A lesão tem repercussão na vida diária afetiva, familiar, social, laboral conforme fotos anteriores e atuais..

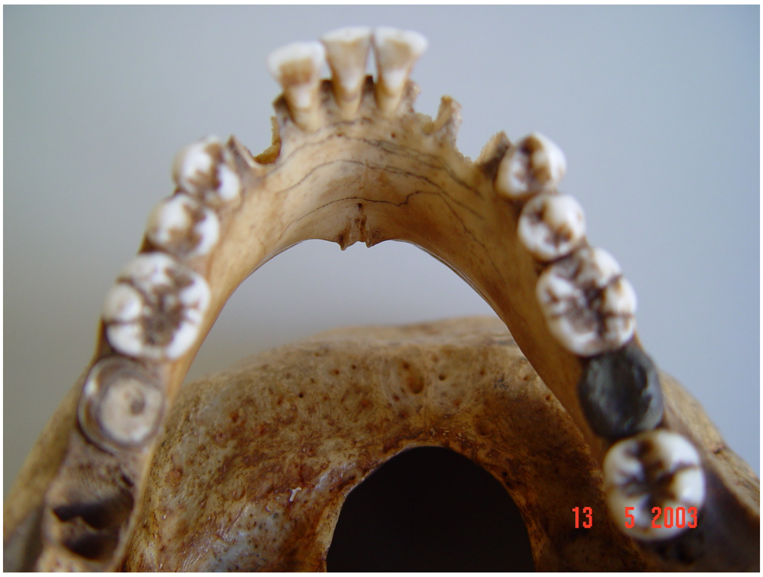

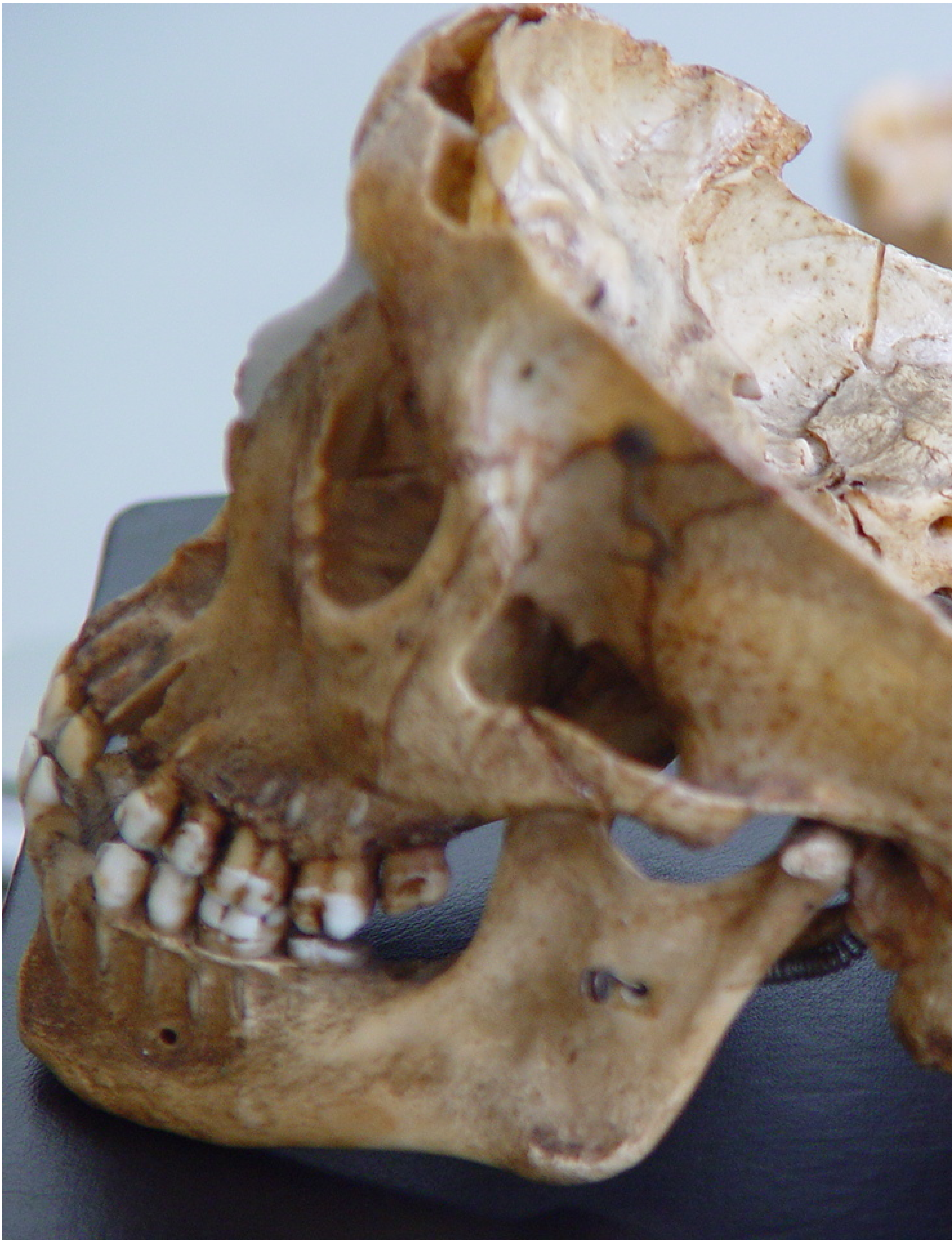

Conclui-se que classificada segundo os parâmetros de dano estético AIPE - Análise do Impacto do Prejuízo Estético (escala de seis níveis e pontuação entre 0 e 50 pontos), como sendo dano estético relativo à área 1, a lesão é caracterizada por 27 pontos e grau de prejuízo estético: Bastante importante.

Escore final: 27;
Grau de prejuízo: Bastante importante.

fousp, 04/07/2017

____

Professor Doutor Nelson Sakaguti
